# ESWT for tendinopathy: technology and clinical implications

**DOI:** 10.1007/s00167-012-2009-3

**Published:** 2012-05-01

**Authors:** Henk van der Worp, Inge van den Akker-Scheek, Hans van Schie, Johannes Zwerver

**Affiliations:** 1 Center for Sports Medicine, University Medical Center Groningen, University of Groningen, P.O. Box 30.001, 9700 RB Groningen, The Netherlands; 2 Department of Orthopaedics, Erasmus University Medical Center, Rotterdam, The Netherlands; 3 Department of Equine Sciences, Faculty of Veterinary Medicine, Utrecht University, Utrecht, The Netherlands

**Keywords:** Tendinopathy, Shockwave, Treatment

## Abstract

**Purpose:**

The general consensus that tendinopathy, at least in the chronic stage, is mainly a degenerative condition and inflammation plays a minor role has led to a shift from treatments that target inflammation towards treatment options that promote regeneration. One of these treatments is extracorporeal shockwave therapy (ESWT), a physical therapy modality that uses pressure waves to treat tendinopathy. This review was undertaken to give an overview of the literature concerning this treatment, and special attention is given to the differences between focused and radial ESWT.

**Methods:**

A narrative description of wave characteristics, generation methods and in vitro effects of ESWT is given. The literature on ESWT as a treatment for one common tendinopathy, patellar tendinopathy, was systematically reviewed.

**Results:**

Waves that are generated for focused and radial ESWT have very different physical characteristics. It is unclear how these characteristics are related to clinical effectiveness. Studies into the biological effects of ESWT have mainly used focused shockwave therapy, showing a number of effects of shockwaves on biological tissue. The systematic review of studies into the clinical effects of ESWT for patellar tendinopathy showed conflicting evidence for its effectiveness.

**Conclusion:**

Physical characteristics of focused and radial waves differ substantially, but effect on clinical effectiveness is unclear. Whereas in vitro studies often show the effects of ESWT on tendon tissue, results of clinical studies are inconsistent. Based on the review of the literature, suggestions are given for the use of ESWT in clinical practice regarding timing and treatment parameters.

**Level of evidence:**

IV.

**Electronic supplementary material:**

The online version of this article (doi:10.1007/s00167-012-2009-3) contains supplementary material, which is available to authorized users.

## Introduction

Tendon injuries (tendinopathies) are common in the entire population, especially in relation to sports and occupation [45, 46]. Tendinopathy has a complex pathophysiology. It consists of a short acute inflammatory stage but after some time, it gradually becomes a degenerative condition [1].

Because both conservative and surgical management of tendinopathy is not always successful, new treatment modalities have been developed. One of these modalities is extracorporeal shockwave therapy (ESWT). In 2002, Chung and Wiley [8] published a review about ESWT for treating tendinopathies. At that time, they concluded based on the literature that there was strong evidence for the effectiveness of ESWT for chronic tendinopathy and that further research was required to settle debates concerning applied energy, number of pulses and number of treatment sessions.

Over the last decade, next to increased knowledge about the pathogenesis of tendinopathy, there have been technical developments and an accumulation of studies examining the working mechanisms of ESWT and its effectiveness. One of the main technical developments is that nowadays two different kinds of ESWT are used for treating tendinopathy: focused ESWT (FSWT) and radial ESWT (RSWT). RSWT is relatively new and has made ESWT more affordable and more widely available. These new technologies are the rationale for this review. Most research has been done using FSWT, but research on RSWT is starting to be published. The aim of the present review is to give an up-to-date description of ESWT, with a special focus on differences between FSWT and RSWT, and review the literature about this treatment method. The overview consists of a description of wave characteristics, methods to generate shockwaves, and in vitro and clinical effects of ESWT, the latter by performing a systematic review with methodological quality assessment on the effects of ESWT for patellar tendinopathy, as an example of a common tendinopathy.

## Pressure waves

**Fig. 1:**
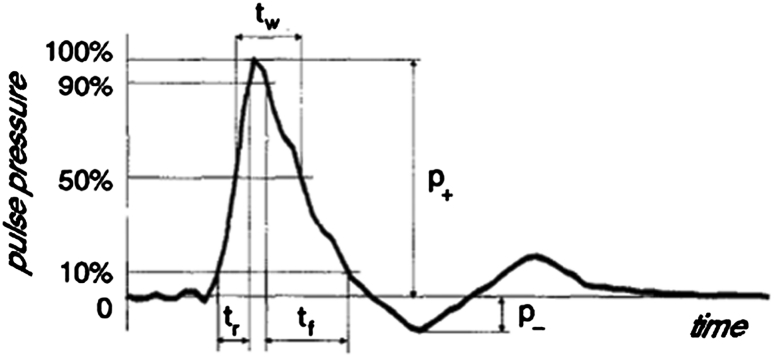
Pressure–time profile of a shockwave (reprinted from [6])

Pressure waves (or sound waves) are oscillating mechanical waves that can travel through gas, liquids and solids. A shockwave is a special, non-linear type of pressure wave (Fig. 1), characterized by a short rise time. The total duration of a shockwave is around 10 μs [10, 42].

Both the positive and the negative phase of a shockwave have an effect on interfaces between tissues with different density (acoustic impedance). During the positive phase, shockwaves with high pressure may hit an interface, leading to reflections, or they may pass and gradually become absorbed. The negative (tensile) phase of the shockwave causes cavitation at the tissue interfaces. During cavitation, air bubbles are formed as a result of the negative pressure. These bubbles subsequently implode with high speed, generating a second wave of shockwaves or micro-jets of fluid [10, 42].

## Types of ESWT

There are two types of shockwave therapy: focused shockwave therapy (FSWT) and radial shockwave therapy (RSWT). This section will describe wave characteristics of both methods.

### FSWT

**Fig. 2:**
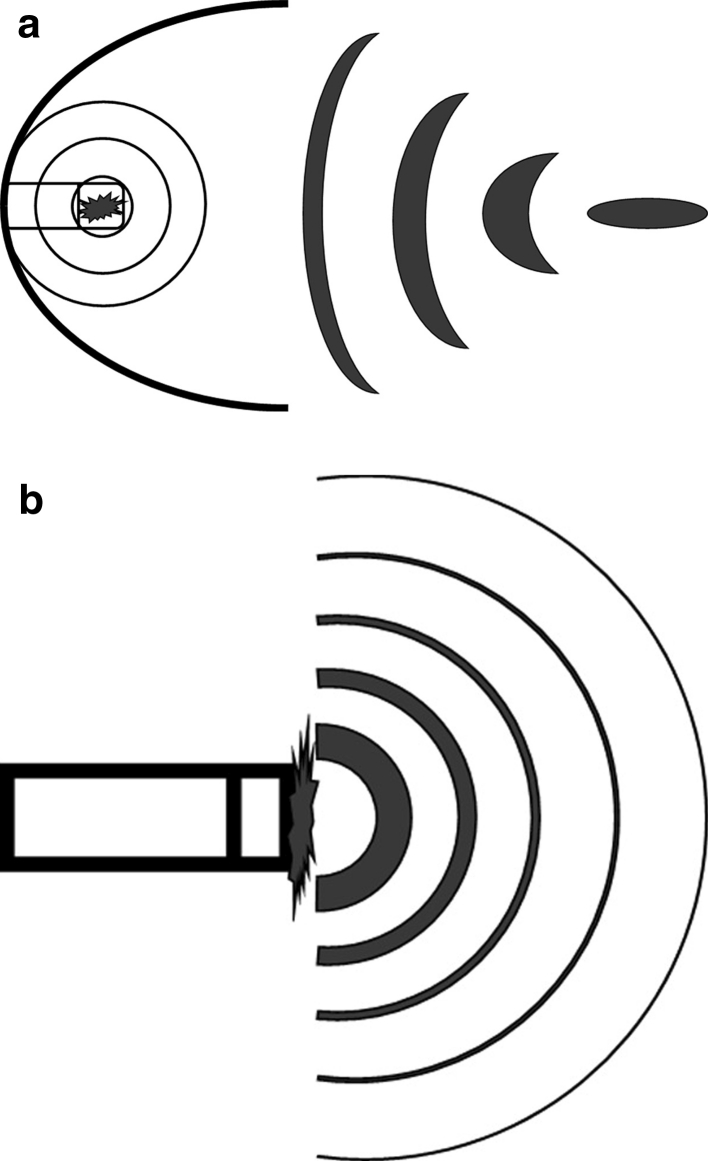
**a** Pressure field of a focused shockwave device (EH-generated by means of spark gap). **b** Pressure field of a radial shockwave device

FSWT is called focused because a pressure field is generated that converges in the adjustable focus at selected depth in the body tissues, where the maximal pressure is reached (Fig. 2a). There are three methods to generate focused shockwaves for FSWT: electrohydraulic (EH), electromagnetic (EM) and piezoelectric (PE) [42]. All three have in common that the waves are generated in water (inside the applicator). Focused shockwaves are generated in water because the acoustic impedance of water and biologic tissue is comparable. As a result of this, reflection is limited and waves are better transferred into the body.

A difference between these three methods is the moment at which the shockwave forms. EH generators produce focused shockwaves at origin, immediately after the spark gap, while EM and PE generators form shockwaves nanoseconds later by means of focusation of waves that are generated [12].

### RSWT

The term radial refers to the diverging pressure field of RSWT devices, which reach a maximal pressure already at the source (Fig. 2b), not at a selected depth in the body. Radial shockwaves for RSWT are generated by accelerating a projectile, using compressed air, through a tube on the end of which an applicator is placed. The projectile hits the applicator and the applicator transmits the generated pressure wave into the body. In contrast to focused shockwave, radial pressure waves are not generated in water.

### FSWT versus RSWT

There are two important differences in wave characteristics between focused shockwaves and radial shockwaves. First, radial shockwaves have a more superficial effect, compared to focused shockwaves, which reach a maximal energy in the focus that is located deeper into the body tissues (Fig. 2) [39]. It was shown that a RSWT device generates a pressure field extending to 40 mm in water, whereas the pressure field generated during FSWT may reach a distance that is about twice as high [39]. How these measures relate to biological tissue is not known. These measures are also dependent of the device that is used and the energy setting. In general, focused shockwaves will travel further and have more impact on deeper located tissues.

**Fig. 3:**
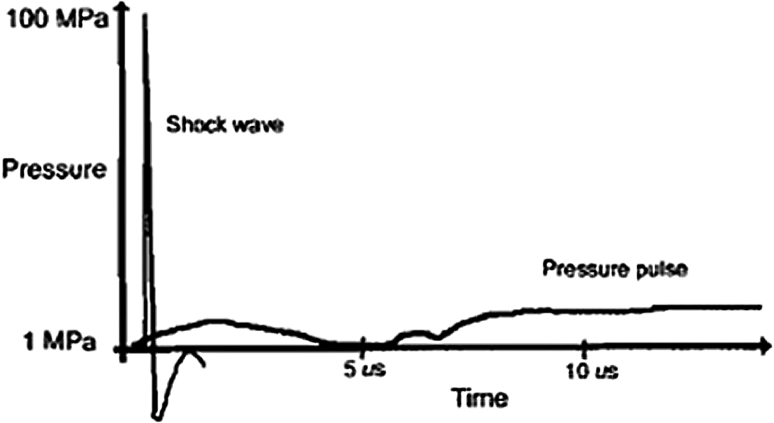
Differences in pressure–time profile of a shockwave (generated with a focused shockwave device) and a pressure wave (generated with a radial shockwave device) (reprinted from [39])

Second, research has shown that pressure waves generated by RSWT from a fundamental point of view cannot be called shockwaves because they lack the characteristic physical features of shockwaves (Fig. 3) such as a short rise time, a high peak pressure and non-linearity [11]. A reason for this is that the speed of sound in tissue is around 1,500 m/s, whereas the projectile during radial pressure wave generation can only reach a speed of around 20 m/s [39]. This speed is not high enough to generate a real shockwave. Chitniss and Cleveland [7] found that the rise time (tr) of the generated wave was 25–40 ns for two focused devices (EH), whereas it was 600 ns for a radial shockwave device. Although 25–40 ns is longer than the definition given above of a shockwave, the waves generated with the EH devices showed the features that are typical for a shockwave (Fig. 1), whereas the wave generated with the radial device lacked these characteristics. Based on these findings, it may be more correct to use the term *radial pressure wave therapy* instead of RSWT. Radial pressure wave devices also come with ‘focused’ applicators. However, Cleveland et al. [11] showed that these applicators do not generate real shockwaves either.

Because it is not clear which wave characteristics generate therapeutic effects, it is difficult to relate physical differences between focused shockwaves and radial pressure waves to clinical effectiveness [9, 36].

## Biological effects of ESWT—in vitro studies

Until now, most fundamental research on ESWT for tendinopathy has been done with focused shockwaves. Fundamental research into the biological effects of ESWT has been concentrated on a number of non-exclusive theories about the working mechanisms of ESWT in tendinopathy. These theories can be roughly divided into pain relief, tissue regeneration and destruction of calcifications.

### Pain relief

Pain relief with ESWT might work by means of hyperstimulation analgesia [40]. Overstimulation of the treated site would lead to a diminished transmission of signals to the brainstem [51]. Animal studies show that ESWT has an influence on pain transmission by acting on substance P [21, 37], calcitonin gene-related peptide (CGRP) expression in the dorsal root ganglion [52] and on neurovascular sprouting [20], Haake et al. however found no effect of ESWT on substance P and CGRP [18].

### Tissue regeneration

A second theory is that ESWT stimulates tissue regeneration. Tissue regeneration by means of ESWT does fit within the framework of mechanotransduction, where mechanical load on the cytoskeleton leads to cell responses and increased protein synthesis [26]. Healthy human tenocytes responded to ESWT with cell growth and increased collagen synthesis [55], mainly type-I, and in affected human tenocytes, ESWT decreased the expression of matrix metalloproteases (MMPs) and interleukins (ILs) that are associated with tendinopathy [19]. Animal studies show that ESWT leads to an increase in collagen production and matrix turnover [3, 4, 23], increased vascularization in the bone–tendon junction [57] and increased tissue regeneration in wound healing and ischaemia [25, 31, 41].

### Destruction of calcifications

Although in vitro studies are lacking, it is thought that ESWT may also destroy calcifications in tendons. This effect is comparable with the way shockwaves are used in lithotripsy to destroy kidney stones. In vivo studies show the disintegration of calcifications in shoulder tendinopathy after ESWT [15, 44].

## Clinical effects of ESWT

Although in vitro studies have demonstrated biological effects of ESWT, clinical effects of ESWT are less clear. In this section, we will focus on patellar tendinopathy, as an example of a common sports injury for which ESWT is increasingly used and which has the same underlying pathology as other common (insertional) tendinopathies [27]. A systematic search of the literature was performed to identify randomized controlled trials (RCTs) that studied the effectiveness of ESWT for patellar tendinopathy. The search was performed in the PubMed and Embase database. Four RCTs were found in this search [43, 53, 56, 58]. The methodological quality of the four indentified studies was independently scored by two authors (Henk van der Worp and Inge van den Akker-Scheek) using the PEDro checklist [35]. Characteristics as well as the PEDro score of the four included studies are shown in Table 1.

**Table 1:** Overview of systematic reviews on the effectiveness of ESWT for tendinopathy

Reference	Number of patients in analysis (total/ESWT group)	ESWT type	Treatment ESWT group	Treatment control group	Follow-up period	Improvement on VISA-P compared to control	Significant difference?	Pedro score
Peers [43]	40/21	FSWT (electromagnetic)	3 ESWT treatments 1-week interval 1,000 impulses 4 Hz 0.2 mJ/mm^2^ Decline squat training	3 placebo ESWT treatments 1-week interval 1,000 impulses 4 Hz 0.03 mJ/mm^2^ Decline squat training	12 weeks	17.4	Yes	8
Taunton et al. [53]	19/10	FSWT (electromagnetic)	3–5 ESWT treatments 1-week interval between treatment 1 and 3 2,000 impulses 0.17 mJ/mm^2^	3–5 placebo ESWT treatments 1 week between treatment 1 and 3 2,000 impulses	12 weeks	3.7	Yes	4
Wang et al. [56]	50/27 (54/30 tendons)	FSWT (electrohydraulic)	1–2 ESWT treatments 1,500 impulses 0.18 mJ/mm^2^	Conservative treatment (NSAIDS, physiotherapy, exercise programme, knee strap and modification of activity levels)	10–53 months	47.6	Yes	5
Zwerver et al. [58]	62/31	FSWT (piezoelectric)	3 ESWT treatments 1-week interval 2,000 impulses 4 Hz 0.25 mJ/mm^2^	3 placebo ESWT treatments 1-week interval 2,000 impulses 4 Hz <0.03 mJ/mm^2^	22 weeks	0.7	No	9

From this table, it appears that, although in vitro studies have demonstrated biological effects of ESWT, the clinical effects of ESWT for the treatment of patellar tendinopathy are less clear. Some studies found ESWT to be effective, whereas in others there was no or little improvement. Remarkably, the study that showed the largest improvement was the only one without a placebo intervention [56].

### Discussion

The most important finding of this review was that there is conflicting evidence regarding the effectiveness of ESWT for patellar tendinopathy. This conflicting evidence may have several reasons. First, there is a lack of objective diagnostic criteria for patellar tendinopathy. Second, it may be that ESWT is only effective during certain stages of tendinopathy and not during other stages. A third reason may be that there are many instrumental settings—like choice of generator (EH, EM or PE), focal depth, number and intensity of pulses (energy flux density)—that can be varied and which may play a role in the effectiveness. A last reason is a methodological one.

These four topics will be described below. These topics are also of importance for research into the effectiveness of other tendinopathies where also conflicting results have been shown [2, 5, 54].

### Diagnosis

There is no gold standard for the diagnosis of tendinopathy. This diagnosis is obtained from a combination of history of symptoms and physical examination [14]. Imaging increases the likelihood of a correct diagnosis, but is not conclusive. The absence of a gold standard may result in non-uniform populations in clinical studies.

### Stage of tendinopathy

Effectiveness of ESWT may depend on the stage of tendinopathy. A recent model of tendinopathy differentiates between a *reactive tendinopathy*/*early tendon disrepair phase* and a *late tendon disrepair*/*degeneration phase* [13]. ESWT seems most appropriate in the latter where the tendinopathy is degenerative and when conservative treatment has no effect [13, 45]. This is also supported by recent studies that showed no effect of ESWT in the early stage of tendinopathy [47, 58]. Until now, studies have not differentiated between subjects in the study based on these different stages; therefore, different studies may have used populations that are not comparable.

### Treatment parameters

There are a number of instrumental settings that can be varied during ESWT (Table 2). The exact relationship between these settings and the effectiveness of the treatment are often unclear, although for some settings there is some indication as to how they may influence effectiveness.

**Table 2:** Treatment parameters

Treatment parameters	Description
Maximal positive pressure	The maximal positive pressure that is reached
Focal zone	A 3-D ellipsoid where the pressure is above a certain value
Energy flux density	The amount of energy/surface unit (mJ/mm^2^)
Time interval between treatments	
Number of impulses/treatment	
Impulse frequency	The number of shockwaves that is applied/second
Localization method	How the to-be-treated site is determined?
Anaesthesia	
Concurrent treatments/rest	

Energy flux densities above 0.50 mJ/mm^2^ should be avoided [38, 51]. Bosch et al. [3] showed in an animal study that EH-generated shockwaves already have a major impact on healthy tendon tissue at an intensity of 0.14 mJ/mm^2^ [3].

Little is known about the optimal number of impulses in tendinopathy, one study showed that three treatments with 500 impulses were more effective than three treatments with 100 impulses in plantar fasciitis [30].

High frequencies do not seem advisable as cavitation bubbles may block the propagation of subsequent waves [10], and the maximum generated pressure seems to drop [12].

Localization of the site that needs treatment can be determined by means of palpation, ultrasound or radiographs. The relationship between these localization methods and pathology is not always clear though [22, 28, 33].

The use of anaesthesia during ESWT seems not advisable as three studies comparing ESWT with and without anaesthesia showed that treatment without anaesthesia is more effective [16, 32, 50].

Rest seems to be important in the first phase after ESWT treatment. Heavy physical activities are best avoided in this phase because the tendon can bear less load shortly after ESWT [3]. This is in line with a recent study that showed no effect of ESWT in actively competing athletes [58]. Although research is scarce, a combination of treatments may have a synergistic effect and lead to better results. Two studies found better results for a combination of ESWT and eccentric exercises than for eccentric exercises alone [43, 48]. Further research on these topics is required.

### Methodology

To prevent that natural improvement, which may be possible in the early stages of tendinopathy, is mistaken for a treatment effect, it is important to include a placebo control group in ESWT effectiveness studies. Furthermore, studies should have a long enough follow-up time to discover treatment effects, since it is known that the metabolic turnover rate of tendon tissue is slow. These methodological issues may also explain some of the conflicting result found for the effectiveness of ESWT (Table 1).

## Clinical effectiveness of FSWT versus RSWT

All four RCTs included in the systematic review on patellar tendinopathy used focused shockwave devices. This may be because radial shockwave devices have been introduced recently. Therefore, no conclusions can be drawn with regard to the effectiveness of RSWT for patellar tendinopathy. For plantar fasciitis, two RCTs have been published that looked at the effect of RSWT [17, 24]. Both studies found RSWT to be effective for this condition. No other placebo controlled studies on the effectiveness of RSWT for treating tendinopathy have been published. There is some evidence from non-placebo controlled studies that RSWT is effective for Achilles tendinopathy [48, 49].

Until now, only one study has directly compared the effectiveness of FSWT and RSWT [34], using both methods to treat plantar fasciitis, and a small difference in favour of FSWT was found. The authors do not hypothesize about what may be the cause of this difference though. Maybe FSWT was more effective because the plantar fascia is located deep in the body (compared to other tendons), so it is better reached with the waves generated by means of FSWT, which achieve their maximal energy within the focus. However, because RSWT also is shown to be effective for treating plantar fasciitis [17, 24], these waves, with a pressure field that reaches around 40 mm in water, probably also travel far enough in tissue to reach the affected area. It is therefore based on the present clinical literature not possible to recommend one of the two types of ESWT over the other.

## Conclusion

Although evidence for the effectiveness of ESWT for treating tendinopathy is inconsistent, it is used widely in sports medicine. The present overview aimed at describing ESWT, in particular the two types that are used: FSWT and RSWT. Waves that are generated for FSWT and RSWT have very different physical characteristics. The relationship between these characteristics and clinical effectiveness is unclear. Studies into the biological effects of ESWT have mainly used FSWT, showing a number of effects of shockwaves on biological tissue. Clinical effects of ESWT for (patellar) tendinopathy are less clear. Reasons for this may be the non-uniform inclusion criteria related to the absence of a diagnostic gold standard, populations from different pathological stages, the large number of treatment parameters that can be varied and methodological issues.

It remains therefore questionable whether ESWT should be recommended at all. This is probably also the case for most other tendinopathies for which also conflicting findings regarding the effectiveness of ESWT have been reported. Further research is required to determine the value of ESWT for tendinopathy. This research should consist of a combination of in vitro and clinical studies. Studies with clear descriptions of study populations, diagnostic criteria and treatment parameters and concurrent rehabilitation programmes/tendon loading activities are necessary to advance research.

### Clinical implications

This review provides some suggestions for the use of ESWT in clinical practice. When ESWT is used to treat tendinopathy, it seems best to apply it in a later stage [13], in combination with tendon load management [29], after other conservative options have been tried and before more radical options like surgery are considered. Based on the literature, low energy, a low frequency, no anaesthetics and exercise after an initial rest period can be recommended. At the moment, no recommendation can be given as to which of the two types of ESWT should be used.

The introduction of RSWT next to FSWT made ESWT more affordable and easier to administer. However, there is no agreement in the literature as to whether ESWT is effective for tendinopathy; hence, at the moment, there is no information available as to which of the two methods is preferable.

## Electronic supplementary material

Below is the link to the electronic supplementary material.

Supplementary material 1 (PDF 258 kb)

Supplementary material 2 (PDF 56 kb)
