# Supplementary material for: ESWT for tendinopathy: technology and clinical implications
Source: Knee Surg Sports Traumatol Arthrosc. 2012 May 1;21(6):1451–8. doi: 10.1007/s00167-012-2009-3 (PMC3657080; doi:10.1007/s00167-012-2009-3)
Supplement: Supplementary file 1 — Supplementary material 1 (PDF 258 kb) [file 167_2012_2009_MOESM1_ESM.pdf]

ELSEVIER LICENSE
TERMS AND CONDITIONS

Sep 15, 2011

This is a License Agreement between Henk van der Worp ("You") and Elsevier ("Elsevier") provided by Copyright Clearance Center ("CCC"). The license consists of your order details, the terms and conditions provided by Elsevier, and the payment terms and conditions.

All payments must be made in full to CCC. For payment instructions, please see information listed at the bottom of this form.

Supplier	Elsevier Limited<br>The Boulevard,Langford Lane<br>Kidlington,Oxford,OX5 1GB,UK
Registered Company Number	1982084
Customer name	Henk van der Worp
Customer address	Hanzeplein 1<br><br>Groningen, other 9713 GZ
License number	2750250116589
License date	Sep 15, 2011
Licensed content publisher	Elsevier
Licensed content publication	Ultrasound in Medicine & Biology
Licensed content title	The performance of different pressure pulse generators for extracorporeal lithotripsy: A comparison based on commercial lithotripters for kidney stones
Licensed content author	A. Buizza,T. dell'Aquila,P. Giribona,C. Spagno
Licensed content date	1995
Licensed content volume number	21
Licensed content issue number	2
Number of pages	14
Start Page	259
End Page	272
Type of Use	reuse in a journal/magazine
Requestor type	author of new work
Intended publisher of new work	Springer Science+Business Media
Portion	figures/tables/illustrations
Number of figures/tables/illustrations	1
Format	both print and electronic
Are you the author of this Elsevier article?	No
Will you be translating?	No

Create PDF files without this message by purchasing novaPDF printer (http://www.novapdf.com)

Order reference number	
Title of the article	ESWT for tendinopathy: technology and clinical implications
Publication new article is in	Knee Surgery, Sports Traumatology, Arthroscopy
Publisher of the new article	Springer Science+Business Media
Author of new article	H. van der Worp
Expected publication date	Jan 2012
Estimated size of new article (number of pages)	6
Elsevier VAT number	GB 494 6272 12
Permissions price	0.00 EUR
VAT/Local Sales Tax	0.0 USD / 0.0 GBP
Total	0.00 EUR
Terms and Conditions	

INTRODUCTION

1. The publisher for this copyrighted material is Elsevier. By clicking "accept" in connection with completing this licensing transaction, you agree that the following terms and conditions apply to this transaction (along with the Billing and Payment terms and conditions established by Copyright Clearance Center, Inc. ("CCC"), at the time that you opened your Rightslink account and that are available at any time at http://myaccount.copyright.com).

GENERAL TERMS

2. Elsevier hereby grants you permission to reproduce the aforementioned material subject to the terms and conditions indicated.

3. Acknowledgement: If any part of the material to be used (for example, figures) has appeared in our publication with credit or acknowledgement to another source, permission must also be sought from that source. If such permission is not obtained then that material may not be included in your publication/copies. Suitable acknowledgement to the source must be made, either as a footnote or in a reference list at the end of your publication, as follows:

“Reprinted from Publication title, Vol /edition number, Author(s), Title of article / title of chapter, Pages No., Copyright (Year), with permission from Elsevier [OR APPLICABLE SOCIETY COPYRIGHT OWNER].” Also Lancet special credit - “Reprinted from The Lancet, Vol. number, Author(s), Title of article, Pages No., Copyright (Year), with permission from Elsevier.”

4. Reproduction of this material is confined to the purpose and/or media for which permission is hereby given.

5. Altering/Modifying Material: Not Permitted. However figures and illustrations may be altered/adapted minimally to serve your work. Any other abbreviations, additions, deletions and/or any other alterations shall be made only with prior written authorization of Elsevier Ltd. (Please contact Elsevier at permissions@elsevier.com)

6. If the permission fee for the requested use of our material is waived in this instance, please be advised that your future requests for Elsevier materials may attract a fee.

Create PDF files without this message by purchasing novaPDF printer (http://www.novapdf.com)

7. Reservation of Rights: Publisher reserves all rights not specifically granted in the combination of (i) the license details provided by you and accepted in the course of this licensing transaction, (ii) these terms and conditions and (iii) CCC's Billing and Payment terms and conditions.

8. License Contingent Upon Payment: While you may exercise the rights licensed immediately upon issuance of the license at the end of the licensing process for the transaction, provided that you have disclosed complete and accurate details of your proposed use, no license is finally effective unless and until full payment is received from you (either by publisher or by CCC) as provided in CCC's Billing and Payment terms and conditions. If full payment is not received on a timely basis, then any license preliminarily granted shall be deemed automatically revoked and shall be void as if never granted. Further, in the event that you breach any of these terms and conditions or any of CCC's Billing and Payment terms and conditions, the license is automatically revoked and shall be void as if never granted. Use of materials as described in a revoked license, as well as any use of the materials beyond the scope of an unrevoked license, may constitute copyright infringement and publisher reserves the right to take any and all action to protect its copyright in the materials.

9. Warranties: Publisher makes no representations or warranties with respect to the licensed material.

10. Indemnity: You hereby indemnify and agree to hold harmless publisher and CCC, and their respective officers, directors, employees and agents, from and against any and all claims arising out of your use of the licensed material other than as specifically authorized pursuant to this license.

11. No Transfer of License: This license is personal to you and may not be sublicensed, assigned, or transferred by you to any other person without publisher's written permission.

12. No Amendment Except in Writing: This license may not be amended except in a writing signed by both parties (or, in the case of publisher, by CCC on publisher's behalf).

13. Objection to Contrary Terms: Publisher hereby objects to any terms contained in any purchase order, acknowledgment, check endorsement or other writing prepared by you, which terms are inconsistent with these terms and conditions or CCC's Billing and Payment terms and conditions. These terms and conditions, together with CCC's Billing and Payment terms and conditions (which are incorporated herein), comprise the entire agreement between you and publisher (and CCC) concerning this licensing transaction. In the event of any conflict between your obligations established by these terms and conditions and those established by CCC's Billing and Payment terms and conditions, these terms and conditions shall control.

14. Revocation: Elsevier or Copyright Clearance Center may deny the permissions described in this License at their sole discretion, for any reason or no reason, with a full refund payable to you. Notice of such denial will be made using the contact information provided by you. Failure to receive such notice will not alter or invalidate the denial. In no event will Elsevier or Copyright Clearance Center be responsible or liable for any costs, expenses or damage incurred by you as a result of a denial of your permission request, other than a refund of the amount(s) paid by you to Elsevier and/or Copyright Clearance Center for denied permissions.

LIMITED LICENSE

Create PDF files without this message by purchasing novaPDF printer (http://www.novapdf.com)

The following terms and conditions apply only to specific license types:

15. **Translation**: This permission is granted for non-exclusive world **English** rights only unless your license was granted for translation rights. If you licensed translation rights you may only translate this content into the languages you requested. A professional translator must perform all translations and reproduce the content word for word preserving the integrity of the article. If this license is to re-use 1 or 2 figures then permission is granted for non-exclusive world rights in all languages.

16. **Website**: The following terms and conditions apply to electronic reserve and author websites:
Electronic reserve: If licensed material is to be posted to website, the web site is to be password-protected and made available only to bona fide students registered on a relevant course if:
This license was made in connection with a course,
This permission is granted for 1 year only. You may obtain a license for future website posting,
All content posted to the web site must maintain the copyright information line on the bottom of each image,
A hyper-text must be included to the Homepage of the journal from which you are licensing at http://www.sciencedirect.com/science/journal/xxxxx or the Elsevier homepage for books at http://www.elsevier.com , and
Central Storage: This license does not include permission for a scanned version of the material to be stored in a central repository such as that provided by Heron/XanEdu.

17. **Author website** for journals with the following additional clauses:

All content posted to the web site must maintain the copyright information line on the bottom of each image, and
he permission granted is limited to the personal version of your paper. You are not allowed to download and post the published electronic version of your article (whether PDF or HTML, proof or final version), nor may you scan the printed edition to create an electronic version,
A hyper-text must be included to the Homepage of the journal from which you are licensing at http://www.sciencedirect.com/science/journal/xxxxx , As part of our normal production process, you will receive an e-mail notice when your article appears on Elsevier's online service ScienceDirect (www.sciencedirect.com). That e-mail will include the article's Digital Object Identifier (DOI). This number provides the electronic link to the published article and should be included in the posting of your personal version. We ask that you wait until you receive this e-mail and have the DOI to do any posting.
Central Storage: This license does not include permission for a scanned version of the material to be stored in a central repository such as that provided by Heron/XanEdu.

18. **Author website** for books with the following additional clauses:
Authors are permitted to place a brief summary of their work online only.
A hyper-text must be included to the Elsevier homepage at http://www.elsevier.com

All content posted to the web site must maintain the copyright information line on the bottom of each image
You are not allowed to download and post the published electronic version of your chapter, nor may you scan the printed edition to create an electronic version.
Central Storage: This license does not include permission for a scanned version of the material to be stored in a central repository such as that provided by Heron/XanEdu.

Create PDF files without this message by purchasing novaPDF printer (http://www.novapdf.com)

19. **Website** (regular and for author): A hyper-text must be included to the Homepage of the journal from which you are licensing at http://www.sciencedirect.com/science/journal/xxxxx. or for books to the Elsevier homepage at http://www.elsevier.com

20. **Thesis/Dissertation**: If your license is for use in a thesis/dissertation your thesis may be submitted to your institution in either print or electronic form. Should your thesis be published commercially, please reapply for permission. These requirements include permission for the Library and Archives of Canada to supply single copies, on demand, of the complete thesis and include permission for UMI to supply single copies, on demand, of the complete thesis. Should your thesis be published commercially, please reapply for permission.

21. **Other Conditions**:

v1.6

Gratis licenses (referencing $0 in the Total field) are free. Please retain this printable license for your reference. No payment is required.

If you would like to pay for this license now, please remit this license along with your payment made payable to "COPYRIGHT CLEARANCE CENTER" otherwise you will be invoiced within 48 hours of the license date. Payment should be in the form of a check or money order referencing your account number and this invoice number RLNK11053072.
Once you receive your invoice for this order, you may pay your invoice by credit card. Please follow instructions provided at that time.

Make Payment To:
Copyright Clearance Center
Dept 001
P.O. Box 843006
Boston, MA 02284-3006

For suggestions or comments regarding this order, contact Rightslink Customer Support: customercare@copyright.com or +1-877-622-5543 (toll free in the US) or +1-978-646-2777.

Create PDF files without this message by purchasing novaPDF printer (http://www.novapdf.com)
